# Fluorescence-Guided Surgery in a Latin American Population: Patient Experiences in a Private Academic Hospital in Panama

**DOI:** 10.7759/cureus.100511

**Published:** 2025-12-31

**Authors:** María Salgado, Roberto Sáenz, Moisés Cukier, Emmy Arrue, Homero Rodríguez-Zentner

**Affiliations:** 1 Department of Medicine, Ciudad de la Salud, Panama City, PAN; 2 Department of General Surgery, Pacifica Salud Hospital, Panama City, PAN

**Keywords:** fluorescence-guided surgery, gastrointestinal surgery, indocyanine green, laparoscopic cholecystectomy, latin america, sentinel lymph node

## Abstract

Introduction: Fluorescence-guided surgery using indocyanine green is a technique used worldwide, with demonstrated benefits such as improved intraoperative visualization of anatomical structures, greater precision in identifying lymphatic drainage, and enhanced assessment of tissue vascularization. This study aimed to describe patients' experiences with fluorescence-guided surgery in a private hospital in Panama.

Methods: A descriptive, observational, retrospective study was conducted between 2019 and 2024 at Hospital Pacífica Salud, Panama.

Results: A total of 432 patients were included: sentinel lymph node mapping in breast cancer (n = 61), gastrointestinal surgeries (n = 97), and gallbladder procedures (n = 274). The mean age was 57 years for sentinel node cases, 56 years for gastrointestinal surgeries, and 46 years for gallbladder surgeries. Among gastrointestinal cases, 89 (92%) were elective, and 8 (8%) were emergencies, with an average blood loss of 113 mL. The ureter was identified through direct visualization in all cases, and no anastomotic leak was recorded. In laparoscopic cholecystectomies, 196 (72%) were elective, and 78 (28%) were emergency procedures, with an average blood loss of 1.9 mL, and no bile duct injuries were reported. For the sentinel lymph node group, successful identification was achieved in 61 (100%) of cases. No procedure-related mortality or allergic reactions were reported.

Conclusion: The experience documented here helps fill the gap in regional data and underscores the need for continued research into its benefits across diverse populations, advancing the goal of more precise, personalized, and safer surgery.

## Introduction

Fluorescence-guided surgery (FGS) is an advanced imaging technique that uses light in the visible, infrared, and ultraviolet spectra to visualize and identify structures that are difficult to detect with the naked eye, as well as to evaluate dynamic processes such as organ perfusion. Although current surgical procedures largely rely on the surgeon’s perception under ambient light, the introduction of fluorescence has revolutionized modern surgical practice by providing a deeper understanding of the anatomy, physiology, and pathology of various anatomical structures [1,2].

Indocyanine green (ICG) is the only fluorescent dye approved by the U.S. Food and Drug Administration (FDA) for this technique. It is characterized by its ability to bind to albumin and distribute uniformly in the bloodstream by attaching to plasma proteins [3]. The liver captures ICG through active transport into hepatocytes, where it is metabolized and excreted almost unchanged in the bile. The uptake of ICG by hepatocytes is highly efficient, generating a marked concentration gradient between the sinusoids and hepatocytes. Once inside the hepatocytes, ICG elimination is limited, and most of the compound is returned to the sinusoids instead of being excreted into the bile. Biliary excretion of ICG is adenosine triphosphate (ATP)-dependent, reflecting both the excretory function and the energetic state of the liver [1].

This mechanism is advantageous because it facilitates the identification of the biliary tract and the visualization of hepatic tumors. Furthermore, indocyanine green absorbs near-infrared (NIR) light and emits fluorescence at a wavelength between 780 and 830 nm. This allows a specialized camera to capture fluorescent images superimposed on visible light, providing surgeons with valuable additional information [1,3]. Advances in minimally invasive techniques have positively impacted short- and medium-term postoperative outcomes; however, surgical complications remain unpredictable and can sometimes be devastating. In this context, FGS has emerged as a promising tool, as it has demonstrated usefulness in reducing various complications through its wide range of applications [4].

There is growing evidence highlighting that FGS is a safe and practical tool, characterized by a short learning curve and multiple aforementioned benefits. However, more studies involving different populations are needed to strengthen the evidence and draw more accurate conclusions, particularly in Latin American cohorts, where reports remain scarce [5,6]. Therefore, documenting the safety and efficacy of this technique through regional experiences, such as the one presented in this study, is of substantial value.

## Materials and methods

Study design and population

A descriptive, observational, retrospective study was conducted at Pacífica Salud Hospital, Panama, between April 2019 and August 2024. Inclusion criteria consisted of patients aged 18 years or older, patients who underwent fluorescence-guided surgery (FGS) in the Department of General Surgery, patients on whom the procedure was performed by a single surgical team during the study period, and patients treated at Pacífica Salud Hospital. Patients with a known allergy to iodine and those with incomplete medical records were excluded from the study. All eligible patients meeting these criteria were included in the analysis.

Procedures and data collection

Data were obtained retrospectively from the institutional database and patient medical records, following the inclusion and exclusion criteria. The information was collected by a team of five investigators using a spreadsheet designed in Microsoft Excel (Microsoft Corporation, Redmond, WA, USA). This spreadsheet functioned as a structured form and contained variables predefined in the study protocol. Access to the dataset was restricted to the investigators and stored in a secure digital folder with a physical backup on an external drive.

Intervention

In all procedures, indocyanine green, a tricarbocyanine fluorophore approved by the U.S. Food and Drug Administration (FDA), was used. In the cholecystectomy group, ICG was administered intravenously during patient preparation, 45 minutes prior to surgery, and before induction of anesthesia. The administered dose consisted of 1 mL of a solution prepared by diluting 25 mg of ICG in 10 mL of sterile water. No intragallbladder injection was performed in any patient. In the sentinel lymph node group, ICG was administered via a retroareolar injection after induction of anesthesia. The administered dose consisted of 1 mL of a solution prepared by diluting 25 mg of ICG in 5 mL of sterile water.

For ureteral visualization, ICG was administered by the urology team via an intraureteral route after ureteral catheterization, with the patient anesthetized and placed in the lithotomy position. The administered dose consisted of 1 mL of a solution prepared by diluting 25 mg of ICG in 10 mL of sterile water. In the gastrointestinal anastomosis group, ICG was administered intravenously at the exact time point when intestinal perfusion needed to be assessed for anastomotic evaluation. The administered dose consisted of 1 mL of a solution prepared by diluting 25 mg of ICG in 10 mL of sterile water.

During surgery, intermittent activation of the NIR light source from the EleVision™ IR Near-infrared (NIR) fluorescence imaging with indocyanine green (ICG) was employed intraoperatively using the EleVision™ IR platform (Medtronic®, Minneapolis, MN, USA), which emits NIR light at 805 nm and allows real-time visualization of anatomical structures and tissue perfusion without causing tissue damage. Depending on the surgical objective, ICG was administered through routes previously described, and fluorescence was subsequently detected using the same NIR-capable platform.

Quality control

Data quality was verified through cross-review by all five investigators before final analysis. Records showing inconsistencies, critical missing data, or failure to meet inclusion criteria were excluded.

Ethical considerations

The study was approved by the Ethics Committee of Pacífica Salud Hospital (approval number 136) and conducted in accordance with the principles of the Declaration of Helsinki. All data were anonymized prior to analysis by removing personal identifiers such as names and medical record numbers. All investigators were certified in Good Clinical Practice (GCP).

Statistical analysis

Data was tabulated in Microsoft Excel®, version 16.0 (Microsoft Corporation, Redmond, WA, USA) and analyzed using Jamovi software, version 2.3.28.0 (The Jamovi Project, Sydney, Australia). Descriptive statistics were applied: means, medians, and standard deviations (SD) for quantitative variables, and absolute and relative frequencies for qualitative variables. Comparative analyses were not performed, as the study was descriptive in nature.

## Results

A total of 432 patients who underwent fluorescence-guided surgery (FGS) at Pacífica Salud Hospital, Panama, between 2019 and 2024 were included. Patients were categorized into three surgical groups based on the type of intervention: sentinel lymph node surgery (n = 61), gastrointestinal surgery (n = 97), and laparoscopic cholecystectomy (n = 274).

Demographic and operative characteristics

General characteristics by surgical group are summarized in Table 1. Patients undergoing the analyzed surgeries showed short operative times, low estimated blood loss, and brief postoperative hospital stays.

**Table 1:** Demographic and operative characteristics by surgical group Summary of patient demographics and key intraoperative variables in three surgical categories: sentinel lymph node (n = 61), gastrointestinal (n = 97), and gallbladder (n = 274). Values are expressed as mean ± standard deviation (SD) for continuous variables and percentages (%) for categorical variables. † Operative time reported as mean minutes. ‡ Estimated blood losses represent the average intraoperative bleeding volume (mL). § Postoperative days indicate the average length of hospital stay. SD = standard deviation; mL = milliliters; min = minutes.

Characteristics	Sentinel Lymph Node (n=61)	Gastrointestinal (n=97)	Gallbladders (n=274)
Age (mean ± SD)	57.0 ± 13.8	56.0 ± 15.0	46.3 ± 13.7
Sex (%)	F: 54 (88.3%)	F: 44 (45.4%)	F: 166 (60.6%)
	M: 7 (11.7%)	M: 53 (54.6%)	M: 108 (39.4%)
Operative time(min) †	16	135	58.9
Estimated blood losses (mL) ‡	20.7	113	1.9
Postoperative days §	0.689	4.54	1.27

Surgical indications

The most common indications for surgery are presented in Table 2. In the sentinel lymph node group, breast cancer was the most frequent diagnosis. In gastrointestinal surgery, diverticulitis and colon cancer predominated. In cholecystectomy, cholelithiasis was the leading indication.

**Table 2:** Most frequent surgical indications by group Distribution of the most common indications for fluorescence-guided procedures in each surgical category. Values in parentheses represent the number of patients per diagnosis. † Each group includes all cases operated on with intraoperative fluorescence imaging using indocyanine green (ICG).

Surgical Group †	Surgery Indication
Sentinel Lymph Node (n=61)	Breast Cancer (53)
	Melanoma (6)
	Ovarian Cancer (1)
	Meckel cells carcinoma (1)
Gastrointestinal (n=97)	Diverticulitis (39)
	Colon Cancer (35)
	Rectum Cancer (5)
	Bowel Obstruction (4)
	Endometriosis (3)
	Acute Abdomen (2)
	Acute Ischemia (2)
	Crohn´s Disease (1)
	Pancreatitis (1)
Gallbladders (n=274)	Symptomatic cholelithiasis (188)
	Cholecystitis (80)
	Gallbladder polyp (3)
	Choledocholithiasis (2)
	Salmonella reservoir (1)

Procedures performed

In the sentinel lymph node group, the most frequent procedure was mastectomy with sentinel lymph node biopsy, representing 12 cases (19.7%), followed by quadrantectomy. In the gastrointestinal group, the following procedures were performed: 40 left colectomies (conventional, laparoscopic, or robotic), 22 right colectomies (conventional, laparoscopic, or robotic), 18 reconnections, four rectal resections, three Hartmann procedures, two transanal resections, one segmental colectomy, one uterine ventrofixation, and one subtotal gastrectomy. Additionally, five intestinal resections were performed for acute abdomen, ischemia, or obstruction.

The ureteral catheterization technique using ICG was applied in colorectal procedures, allowing continuous visualization of the ureteral course and minimizing the risk of iatrogenic injury. Notably, no ureteral injuries occurred in this cohort. In the cholecystectomy group, all cases were laparoscopic procedures, performed for acute cholecystitis or symptomatic choledocholithiasis. Intraoperative fluorescent cholangiography was used to enhance the identification of the biliary anatomy.

Elective versus emergency procedures

**Figure 1:**
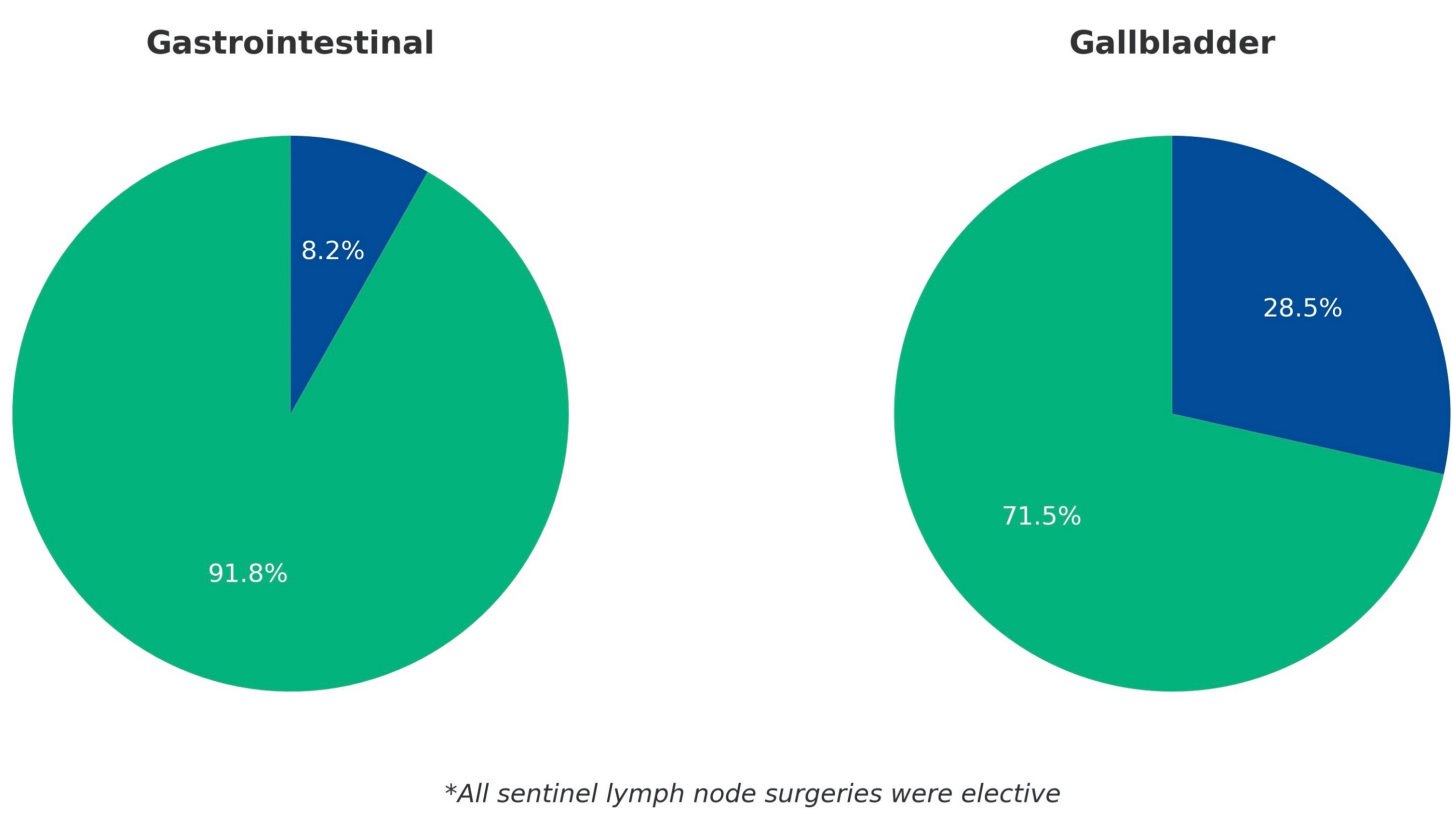
Proportion of elective vs emergency surgeries by surgical group Pie charts show the relative distribution of elective and emergency cases within each surgical category. All sentinel lymph node procedures were elective. Among gastrointestinal surgeries, 89 (91.8%) were elective and eight (8.2%) were emergency procedures. In gallbladder surgeries, 196 (71.5%) were elective and 78 (28.5%) were performed in emergency settings.

The distribution of elective and emergency cases by group is shown in Figure 1. All sentinel lymph node procedures were elective, whereas approximately one in four cholecystectomies was performed as an emergency procedure.

Postoperative complications

**Figure 2:**
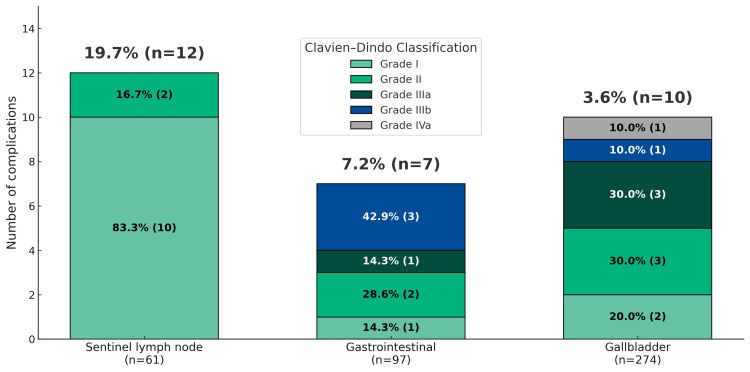
Postoperative complication across surgical groups Stacked bars represent the number and percentage of postoperative complications across three surgical groups: sentinel lymph node (n = 61), gastrointestinal (n = 97), and gallbladder (n = 274). The classification grades complications by increasing severity: Grade I = minor deviation from normal postoperative course, Grade II = pharmacologic treatment required, Grade IIIa = surgical/interventional management without general anesthesia, Grade IIIb = reoperation under general anesthesia, and Grade IVa = life-threatening complication requiring Intensive Care Unit care. The numbers and percentages shown within each bar segment correspond to the proportional distribution of each complication grade relative to the total number of complications for that surgical group.

Postoperative complications are detailed in Figure 2. The majority of patients experienced no adverse events. The most relevant complications included axillary seromas in breast surgery, surgical site infections in gastrointestinal cases, and abdominal collections after cholecystectomy. No allergic reactions to ICG or procedure-related mortality were reported.

Summary of outcomes

Across all groups, FGS with indocyanine green demonstrated a favorable safety profile, with low complication rates and no procedure-related mortality or allergic events. The use of near-infrared fluorescence imaging enhanced intraoperative visualization and supported real-time surgical decision-making in diverse scenarios, including oncologic, gastrointestinal, and biliary surgery. Additionally, we highlight a 100% rate (61 cases) of identification of sentinel lymph nodes with fluorescence as an isolated technique, no urethral lesions, and no bile structure lesions in these large cohorts.

## Discussion

This study represents one of the first large Latin American series on fluorescence-guided surgery applied transversally across general surgery, demonstrating that consistent, high-safety outcomes are achievable even in resource-adapted environments. In this single-center experience from a private hospital in Panama, we achieved a 100% (61 cases) sentinel lymph node detection rate, no urethral lesions in 97 gastrointestinal surgeries, no bile duct injuries in 274 laparoscopic cholecystectomies; with no adverse reactions to ICG and no procedure-related mortality. These results highlight the feasibility and effectiveness of fluorescence-guided surgery in real-world Latin American surgical practice; and are comparable to smaller cohorts previously reported by this group [4,7]. Our results align with the global Delphi consensus published in Annals of Surgery by Dip et al., in which international experts agreed that FGS is both highly effective and very safe across a wide range of procedures [8]. No adverse events or mortality occurred in our cohort, echoing the conclusion that this technique has a strong safety profile. This consistent absence of complications reinforces the reproducibility of FGS safety in Latin American healthcare systems, confirming that the use of ICG can be generalized beyond high-income regions.

The 100% detection rate of sentinel lymph nodes (61 cases) reported here lies at the upper limit of what has been documented in prior series using ICG and fluorescence techniques for lymphatic mapping. The article “Use of fluorescence imaging during lymphatic surgery” (Dip et al., 2022) does not report a specific sentinel node identification rate in breast cancer, as its focus is broader (fluorescence use in lymphatic and lymphedema surgery). This finding is consistent with international literature, which indicates that the ICG technique enables precise, real-time visualization of critical structures, such as sentinel lymph nodes and blood vessels, thereby improving surgical accuracy and the identification of affected areas [9]. Nevertheless, the authors noted that intraoperative visualization of sentinel nodes is among the most promising applications of the technique [10]. Other, more procedure-specific studies have reported variable detection rates depending on the dose, timing of injection, imaging equipment, and surgical team experience, with most series showing high performance or slightly lower results during early adoption phases [11,12].

In our series of 97 gastrointestinal surgeries, ureteral visualization combined with intraoperative perfusion assessment helped prevent ureteral injuries; no ureteral injuries were recorded. This finding is notable and warrants comparison with published experiences of intraoperative fluorescence in colorectal surgery [4,5]. The European Association for Endoscopic Surgery (EAES) conducted a consensus review that systematically analyzed studies from PubMed, Embase, and Cochrane to identify evidence on the potential benefits of fluorescence-guided surgery.

Regarding perfusion assessment and anastomotic leak prevention, the use of ICG was found to be associated with a decreased incidence of anastomotic leaks, particularly in rectal surgery (relative risk (RR) = 0.32, 95% CI 0.22-0.49, p < 0.01, I² = 0%) [13]. Our findings mirror the Delphi Colorectal Consensus led by Wexner et al., in which experts mostly agreed that FGS significantly reduces the incidence of anastomotic leaks through real-time perfusion assessment [14]. In our 97 gastrointestinal procedures, including 90 colorectal cases, no anastomotic leaks were recorded, a result consistent with international data supporting ICG’s capacity to identify ischemic areas before transection. The Delphi experts further emphasized that fluorescence should be applied particularly in left-sided anastomoses; however, our data show successful implementation across both elective and emergency colorectal surgeries, highlighting its adaptability and safety even in urgent settings. Furthermore, the consensus established that the optimal ICG dose ranges between 5-10 mg administered 30-60 seconds prior to perfusion assessment [14]. Our standardized protocol of 2.5 mg/mL (1 mL) administered 45 minutes before surgery provided equally reliable visualization, suggesting that simplified low-dose regimens may be sufficient when using modern NIR platforms. These results reinforce the notion that procedural reproducibility and safety are attainable even with resource-adapted protocols. Therefore, there is a strong recommendation for the use of fluorescence imaging in colorectal surgery to evaluate tissue perfusion and thereby reduce the risk of anastomotic leakage, which is consistent with our results, as no such complications occurred in any of the cases included in our series [10,13].

The retrospective study by Rogers et al. on the use of indocyanine green fluorescence in colorectal surgery highlighted its effectiveness for the intraoperative identification of the ureters [15]. This technique significantly improved ureteral visualization, reducing the risk of associated injuries and complications. The results suggested that fluorescence contributes to greater procedural safety by helping prevent ureteral damage [15].

The study by Polom et al. employed dual-fluorophore imaging (ICG for angiography and methylene blue (MB) for ureteral visualization) during colorectal resections, aiming to simultaneously assess perfusion and ureteral localization. In their cohort, ureteral visualization was achieved in 11 of 12 patients (91.6%), while ICG angiography was successful in all cases. They also reported an iatrogenic ureteral injury rate of 0.4% across their broader colorectal series, corresponding to approximately one case in the entire cohort [3]. The absence of any ureteral injury in our larger cohort underscores the preventive potential of systematic fluorescence use for simultaneous perfusion evaluation and ureteral delineation.

In our cholecystectomy series, fluorescence cholangiography proved particularly useful even in acute settings, with the remarkable finding that no bile duct injuries occurred in any of the operated cases. This zero-injury rate in our 274 laparoscopic cholecystectomies is clinically significant and suggests a protective benefit from real-time visualization of biliary anatomy. Our outcomes parallel those of the International Delphi Survey on Fluorescent Cholangiography, which involved 28 experts and reached a strong consensus that near-infrared fluorescent cholangiography (NIFC) improves biliary anatomy identification and should be used routinely [16].

For comparison, Morales-Conde et al. reviewed the use of ICG fluorescence in general surgery, emphasizing its intraoperative cholangiographic application in cholecystectomy. They noted that fluorescence cholangiography enables real-time visualization of extrahepatic biliary structures, facilitating dissection and reducing the risk of bile duct injury (BDI) [6]. Although Morales-Conde and colleagues did not provide a pooled quantitative rate of BDI in the reviewed literature, they emphasized that distorted anatomy, obesity, or acute inflammation can still make fluorescence interpretation challenging and carry residual risk.

In a retrospective study evaluating the use of ICG cholangiography in 65 patients, the technique allowed identification of the cystic duct (CD) in 54 cases (83.1%). It is noteworthy that in the remaining 11 cases (16.9%), where the CD could not be identified, patients exhibited higher levels of inflammation, evidenced by elevated white blood cell counts and C-reactive protein (CRP) levels, which were also associated with longer operative times and higher conversion rates to open surgery. Furthermore, patients with acute cholecystitis experienced greater difficulty in identifying both the CD and the common bile duct (CBD), further complicating the surgical procedure [17].

When contextualized across surgical subspecialties, our findings also align with the two large multicentric Delphi consensuses covering different surgical fields, where most experts agreed that fluorescence imaging improves anatomical visualization, intraoperative confidence, and decision-making, with a relatively short learning curve [8,18]. In our institution, this technology has enhanced procedural safety and improved decision-making.

The Annals of Surgery consensus also reached full agreement that fluorescence imaging does not increase the overall perioperative cost of care and may reduce costs through shorter operative times and fewer complications [8]. Our experience supports this statement: in more than 400 cases, FGS contributed to zero bile duct injuries, zero ureteral injuries, no conversions, and minimal blood loss, outcomes that translate directly into economic savings and reduced postoperative morbidity. These findings are particularly relevant in middle-income regions such as many Latin American countries, where the balance between innovation and costs is critical for sustainable healthcare delivery.

Despite the high degree of global consensus regarding its safety and efficacy, the need for standardization remains a common theme across all Delphi surveys. Both the Annals of Surgery and Surgery publications emphasize the importance of establishing multicenter registries and performing prospective comparative studies to define the optimal dosing, timing, and fluorescence intensity thresholds across different surgical applications [8,18]. Our experience contributes real-world evidence from a Latin American tertiary setting, demonstrating that consistent results can be achieved with standardized, low-cost protocols. Future collaborative research should focus on cost-effectiveness analyses, training outcomes, and patient-reported metrics to further strengthen the evidence base for fluorescence-guided surgery in our region.

One of the main strengths of our study is that it is among the first large Latin American series to evaluate the cross-disciplinary applicability of fluorescence-guided surgery with ICG in breast, gastrointestinal, and biliary procedures within a single hospital. This grants the study pioneering and regional value, providing data from a population largely underrepresented in international research. Furthermore, our findings demonstrate that FGS can be implemented safely, reproducibly, and with consistent results, even outside high-volume academic centers. We achieved full sentinel lymph node identification, complete ureteral visualization without injury in 97 gastrointestinal surgeries, and no bile duct injuries during cholecystectomy-results that reflect strong clinical effectiveness and reinforce the preventive role of fluorescence in reducing major surgical complications.

Another important strength is the controlled heterogeneity of the clinical settings, which allowed us to demonstrate the versatility of FGS in both elective and emergency contexts, across different surgical subspecialties. This supports the broad applicability of the technique in tertiary-care hospitals. Finally, the absence of adverse reactions to ICG strengthens its safety profile in our population and confirms its role as a reliable, non-toxic imaging adjunct.

However, this study also presents limitations inherent to its cross-sectional observational design, which precludes establishing causal relationships between fluorescence use and complication reduction. The absence of a control group using conventional techniques limits direct comparison of relative efficacy. Selection bias may be present as cases were performed by a single experienced surgical team.

## Conclusions

Overall, fluorescence-guided surgery with indocyanine green proved to be a safe, versatile, and effective tool in diverse surgical scenarios, including breast, gastrointestinal, and biliary procedures. Its use enabled more precise anatomic visualization, contributing to the prevention of major complications, with consistent sentinel node identification, complete ureteral visualization without injuries, and no bile duct injuries reported. These findings support the routine implementation of fluorescence imaging as an intraoperative aid in general surgery and highlight the need for multicenter comparative studies to further evaluate its clinical impact and cost-effectiveness in Latin American populations.
